# Evaluating the use of multimedia information when recruiting adolescents to orthodontics research: A randomised controlled trial

**DOI:** 10.1177/14653125211024250

**Published:** 2021-07-06

**Authors:** Peter Knapp, Nicky Mandall, Wendy Hulse, Jenny Roche, Thirimon Moe-Byrne, Jacqueline Martin-Kerry, Rebecca Sheridan, Steven Higgins, (for the TRECA study group)

**Affiliations:** 1 Department of Health Sciences & the Hull York Medical School, University of York, York, UK; 2 Tameside General Hospital, Ashton-under-Lyne, Tameside, UK; 3 Department of Health Sciences, University of York, York, UK; 4 University of Durham, Durham, UK

**Keywords:** trial, adolescents, research ethics, multimedia information, consent

## Abstract

**Objective:**

To compare two methods of providing information about the Bone Anchored Maxillary Protraction (BAMP) trial: standard printed information and multimedia websites, for their quality and ease of understanding, and impact on decision-making.

**Design:**

Randomised controlled trial.

**Setting:**

Orthodontic outpatient clinic in the UK.

**Methods:**

Participants were 109 adolescents (aged 11–14 years) attending for orthodontic treatment. While awaiting treatment they were asked to imagine being recruited to the BAMP clinical trial. They were individually randomised to receive the printed or the multimedia website information (comprising text, animations and ‘talking head’ videos). After reading or viewing the information, they completed a 9-item Likert scale Decision-Making Questionnaire (DMQ) (score range 0–36) plus three free-text questions on their evaluation of the information.

**Results:**

A total of 104 participants completed the questionnaire. Mean total DMQ scores were higher (more positive) in the website group (28.1 vs. 27.0), although the difference was small and not statistically significant (*P* = 0.20). Analysis of individual questionnaire items showed two statistically significant differences: the website information had higher ratings on ‘easy to understand’ (Z = 3.03; *P* = 0.003) and ‘confidence in decision-making’ (Z = 2.00; *P* = 0.044). On the three free-text questions, more positive and fewer negative comments were made about the websites than the printed information.

**Conclusion:**

In this hypothetical trial setting, adolescent patients found that trial information conveyed on a multimedia website was easier to understand and made them more confident in their decision about trial participation. Their subjective evaluations of the website were also more positive and less negative than about the printed information. Multimedia information has the potential to increase the quality of engagement and information exchange when seeking consent for research.

## Introduction

Randomised controlled trials (RCTs) are the best way to evaluate the effectiveness and safety of healthcare interventions, although around half of trials fail to recruit to time and target, causing delays and increased costs (McDonald et al., 2006; Treweek et al., 2018c). Poor recruitment can lead to underpowered and inconclusive trials (Treweek et al., 2018c).

Potential trial participants must be provided with information, allowing them to make an informed decision on participation, and a recent ‘review of reviews’ reported that participant information can both facilitate and impede recruitment (Sheridan et al., 2020a). The information should provide a thorough understanding of what the research entails. However, printed trial information has been criticised repeatedly as being too long and technical, hard to navigate and not engaging (Caldwell et al., 2012; Eder et al., 2007).

These problems may be magnified in trials involving children or adolescents, who should have an opportunity to understand what the research entails and, when appropriate, participate in decision-making (Nuffield Council on Bioethics, 2015). However, it may be more difficult for them to understand relevant concepts and terms, or the implications of participation (Barfield and Church, 2005; Simon et al., 2004; Stryker et al., 2005; Tait et al., 2015a). In particular, children and young people may struggle to understand procedures and risks (Hunfeld and Passchier, 2012).

Depending on the young person’s age and maturity, and the family dynamics, decisions on trial participation may follow discussion with their family; this may increase the negative effects of difficult or unclear information. A recent review highlighted the importance of providing research information to children and adolescents directly, not just via parents, and stressed that it should be both ‘appealing and understandable’ (Crane and Broome, 2017). However, it is important that this information, used to inform consent or assent decisions, should not be marketing, nor prioritise entertainment at the expense of its information function.

The UK Health Research Authority recently recommended exploration of the use of non-print media for potential research participants (Health Research Authority, 2016). A novel approach is to use multimedia information, whether as a website or offline, allowing written information to be replaced by, or presented alongside, animations, videos, audio and infographics. Multimedia information may increase comprehension of medical information compared with traditional paper-based formats (Hermann, 2002; Hopper et al., 1994; Tait et al., 2009c, 2012b), potentially through enhanced choice and flexibility, increased engagement, and allowing the user to access information content in a non-linear way. Several studies suggest that multimedia websites can help to inform and recruit potential research participants (Hutchison et al., 2007; Tait et al., 2015a; Tait and Voepel-Lewis, 2015d) although none of these studies included children or adolescents. In part, multimedia websites offer great potential as a platform for mandated health communication because people are increasingly familiar with obtaining health and other information digitally (Antoniou et al., 2011; Shneerson et al., 2012). However, it is not clear that everyone prefers digital or online information; some may prefer traditional printed materials. Furthermore, access to digital information and technology is not universal, and unequal access may compound existing income-related health inequalities (Office for National Statistics, 2019). In addition, a recent systematic scoping review highlighted important concerns that children and adolescents have about digital health technologies, including privacy and trustworthiness (Blower et al., 2020).

The TRECA (TRials Engagement in Children and Adolescents) study is evaluating the effectiveness of multimedia websites compared to printed information, when recruiting children and adolescents to trials (Martin-Kerry et al., 2017). This is being undertaken through a linked series of studies within a trial (SWATs), to compare the effects of two information formats (the REC-approved printed information sheets for participants [ISPs], and multimedia websites [MMIs]) on patient recruitment and decision-making (Rick et al., 2014; Treweek et al., 2018a, 2018b, 2018c). One of the included SWATs is the bone anchored maxillary protraction (BAMP) trial (Mandall, 2014).

After the small BAMP trial and its linked SWAT had closed to recruitment, there was an opportunity to evaluate the two forms of trial information with a larger number of adolescents awaiting orthodontic treatment, who were not being recruited to the BAMP trial itself. During this process, we asked them to imagine being asked to take part in the BAMP clinical trial. The aim of the present study was to compare the multimedia websites and printed information for their quality and ease of understanding, and their impact on decision-making.

## Methods

### Study design

The study used a two-arm, parallel-group, individually randomised controlled trial design. Participants were asked to imagine being approached about participation in the BAMP trial of orthodontic treatment (Mandall, 2014).

Participants were randomised to receive a printed participant information sheet (ISP) or view the multimedia website (MMI). The allocation sequence was generated by the TRECA team at the University of York, using block sizes of six allocations and a random number generator (Sealed envelope, 2019). The allocations were provided to the recruitment site in sequential sealed opaque envelopes.

### Study participants

Participants were patients aged 11–14 years and attending the orthodontic clinic for routine appointments at Tameside and Glossop NHS Trust in the UK.

### Interventions

The printed ISP was the standard participant information sheet used in the BAMP trial, which had been approved by NHS Research Ethics Committee and comprised 2276 words across seven printed A4 pages (see Supplementary data). The ISP text addressed the patient (e.g. ‘your treatment’ rather than ‘their treatment’).

The multimedia website (MMI) for the BAMP trial, which was viewed on a tablet computer, had been developed by the TRECA research team and Morph, a website creation company. It contained all the ISP written content, with the text amended to improve clarification when required. The website text addressed the patient (e.g. ‘your treatment’ rather than ‘their treatment’). The website also included five short animation videos, each lasting 45–60 s (‘Summary of the key aspects of the BAMP trial’; ‘Why do we do trials?’; ‘What are trials?’; ‘Who’s in a trial team’; ‘Assent and consent’), and 17 short single-person ‘talking head’ videos, featuring three individuals (10 with the trial principal investigator, four with an adolescent who had received bone anchored maxillary protraction, three with a parent of a child who had received bone anchored maxillary protraction), each lasting 15–50 s and describing different aspects of the trial and clinical procedures. The website content was organised on six main webpages with the following headings: ‘Home page (including summary animation)’; ‘About the trial’; ‘Taking part’; ‘After the trial’; ‘Questions’; and ‘Contacts’ (see Morph (2020) for the link to examples of BAMP MMI content).

The TRECA study websites drew on extensive underpinning qualitative research (Martin-Kerry et al., 2019) and user testing (Sheridan et al., 2019), and were informed by principles of Plain English and information design (Knapp et al., 2011), as well as age-appropriateness and readability formulae (Readabilityformulas.com). The TRECA Patient and Public Involvement Group commented on the content and design of the websites throughout their development (Sheridan et al., 2020b).

### Procedure

Adolescents attending for treatment were asked by the orthodontist to take part in the study while they were seated in the waiting area. After giving written assent (counter-signed by a parent), they were randomly allocated to receive either the printed information or the website presented on a tablet computer. Participants had as long as they needed to read or view the information, usually 15–20 min, after which they were given a printed Decision-Making Questionnaire (DMQ), which they completed immediately after accessing the trial information. Parents attending with the adolescent patient could also read or view the information (as preferred), and also complete the DMQ with them.

### Outcome measure

The primary outcome of this trial was the total score derived from the nine-item decision-making questionnaire (DMQ) (Table 1), which asked respondents to rate different aspects of the information and its impact on decisions. On the first item, respondents evaluated ease of understanding (with five response choices ranging from ‘very hard’ to ‘very easy’); on the other eight items, they were asked to state their level of agreement with statements about the information (with five response choices ranging from ‘not at all’ to ‘yes, completely’). Each of the nine Likert scale items was scored 0–4, deriving a maximum scale score of 36. A higher DMQ score indicates better quality of decision-making. The DMQ comprised items evaluating aspects of trial participation decision-making indicated as important in the underpinning empirical work (Martin-Kerry et al., 2017, 2019; Sheridan et al., 2019, 2020b), including items on: information content; the experience of participation; uncertainty in trials; participation advantages and disadvantages; the process of decision-making; and decisional confidence. The nine scale items were followed by three free-text questions that asked respondents: to suggest any additional information they would have wanted; to identify aspects that were explained well; and for any other comments.

**Table 1.** Decision-Making Questionnaire item responses, by trial allocation.

		Very hard	Hard	OK	Easy	Very easy	Missing
1. The information I saw about the BAMP trial was easy to understand.	MMI	0 (0.0)	1 (1.9)	8 (14.8)	30 (55.6)	13 (24.1)	2 (3.7)
	ISP	0 (0.0)	2 (3.6)	20 (36.4)	25 (45.5)	5 (9.1)	3 (5.5)
	Overall	0 (0.0)	3 (2.8)	28 (25.7)	55 (50.5)	18 (16.5)	5 (4.6)
		Not at all	Not really	Not sure	Yes, mostly	Yes, completely	Missing
2. After seeing the information about the BAMP trial I knew what taking part would be like.	MMI	0 (0.0)	0 (0.0)	7 (13.0)	30 (55.6)	15 (27.8)	2 (3.7)
	ISP	0 (0.0)	1 (1.8)	8 (14.6)	27 (49.1)	16 (29.1)	3 (5.5)
	Overall	0 (0.0)	1 (0.9)	15 (13.8)	57 (52.3)	31 (28.4)	5 (4.6)
3. The information helped me understand how my treatment or care might change if I took part in the BAMP trial.	MMI	0 (0.0)	2 (3.7)	5 (9.3)	31 (57.4)	14 (25.9)	2 (3.7)
	ISP	0 (0.0)	3 (5.5)	4 (7.3)	33 (60.0)	11 (20.0)	4 (7.3)
	Overall	0 (0.0)	5 (4.6)	9 (8.3)	64 (58.7)	25 (22.9)	6 (5.5)
4. The possible benefits of taking part in the BAMP trial were made clear in the information.	MMI	0 (0.0)	1 (1.9)	9 (16.7)	19 (35.2)	23 (42.6)	2 (3.7)
	ISP	0 (0.0)	2 (3.6)	4 (7.3)	29 (52.7)	17 (30.9)	3 (5.5)
	Overall	0 (0.0)	3 (2.8)	13 (11.9)	48 (44.0)	40 (36.7)	5 (4.6)
5. The possible disadvantages of taking part in the BAMP trial were made clear in the information.	MMI	0 (0.0)	5 (9.3)	6 (11.1)	20 (37.0)	21 (38.9)	2 (3.7)
	ISP	0 (0.0)	2 (3.6)	11 (20.0)	24 (43.6)	15 (27.3)	3 (5.5)
	Overall	0 (0.0)	7 (6.4)	17 (15.6)	44 (40.4)	36 (33.0)	5 (4.6)
6. The information about the BAMP trial helped me discuss the trial with the person who asked me to take part (usually a doctor, nurse or researcher).	MMI	1 (1.9)	4 (7.4)	10 (18.5)	23 (42.6)	14 (25.9)	2 (3.7)
	ISP	0 (0.0)	0 (0.0)	12 (21.8)	31 (56.4)	9 (16.4)	3 (5.5)
	Overall	1 (0.9)	4 (3.7)	22 (20.2)	54 (49.5)	23 (21.1)	5 (4.6)
7. The information about the BAMP trial helped me discuss taking part with my parent(s) or family.	MMI	1 (1.9)	1 (1.9)	6 (11.1)	32 (59.3)	12 (22.2)	2 (3.7)
	ISP	0 (0.0)	1 (1.8)	12 (21.8)	26 (47.3)	13 (23.6)	3 (5.5)
	Overall	1 (0.9)	2 (1.8)	18 (16.5)	58 (53.2)	25 (22.9)	5 (4.6)
8. I am confident that I have made the right decision about whether or not to take part in the BAMP trial.	MMI	0 (0.0)	3 (5.6)	4 (7.4)	22 (40.7)	23 (42.6)	2 (3.7)
	ISP	1 (1.8)	0 (0.0)	11 (20.0)	27 (49.1)	13 (23.6)	3 (5.5)
	Overall	1 (0.9)	3 (2.8)	15 (13.8)	49 (45.0)	36 (33.0)	5 (4.6)
9. In all, the information about the BAMP trial helped me make my decision about whether or not to take part.	MMI	0 (0.0)	1 (1.9)	5 (9.3)	24 (44.4)	22 (40.7)	2 (3.7)
	ISP	0 (0.0)	0 (0.0)	7 (12.7)	31 (56.4)	14 (25.5)	3 (5.5)
	Overall	0 (0.0)	1 (0.9)	12 (11.0)	55 (50.5)	36 (33.0)	5 (4.6)

Values are given as n (%).

BAMP, bone anchored maxillary protraction; ISP, participant information sheet; MMI, multimedia website.

Secondary outcomes were the nine individual questionnaire item scores, and the free-text responses to three questions, including the frequency of positive and negative comments about the information.

### Masking

Participants could not be masked to allocation, as they were aware of the information format they received. However, they had no access to the printed or website information that they had not been allocated to receive. The recruiting researchers could not be masked to the trial allocation but had no influence on participants’ responses.

### Sample size

We estimated that a sample size of 109 would give 90% power (alpha 0.05) to detect a statistically significant difference between the groups. This allowed for 10% of participants not completing the questionnaires. We assumed that a mean between-groups difference of 4.5 on the total score (reflecting a mean of 0.5-point difference on each of the nine Likert questions) would be meaningful, and estimated that the standard deviation (SD) of the pooled scores would be 6.75 (assuming that 95% scores would fall in the range of 4.5–31.5).

### Statistical and free-text question analyses

Analyses were conducted using Stata v16 (StataCorp, 2019) on an intention-to-treat basis, using two-sided tests at the 5% significance level. When two adjacent scores for a questionnaire item were given by the participants, the lower score was included. Up to three missing values on items 1–9 were allowed per participant, with a total score calculated by replacing the missing values with the mean score from the completed responses given by the participant.

Total DMQ scores were compared between the ISP and MMI groups using a linear regression, where total DMQ score was the dependent variable and TRECA allocation and gender were independent variables. A sensitivity analysis was conducted in which the analysis was repeated including only participants that had responses to all nine DMQ questions. Adjusted mean differences (AMDs) are presented alongside 95% confidence intervals (CIs) and *P* values. Model assumptions were checked.

Further analyses were conducted to assess the differences in scores on each question between the two groups. Scores were compared using Wilcoxon Mann–Whitney tests (medians, interquartile ranges [IQRs], z-statistics and *P* values are presented). There is an increased risk of Type I error due to multiple testing.

Answers to the free-text questions were analysed statistically (according to the number of respondents making positive or negative evaluations of the information) and descriptively (using a basic content analysis). An odds ratio comparing the number of positive responses between the two groups is presented alongside a 95% CI and *P* value.

Questionnaire data were inputted by one researcher and a random 10% data check was undertaken by a second researcher.

### Ethical approval

Research ethics approval for this study was received from the Yorkshire & the Humber – Bradford Leeds Research Ethics Committee (17/YH/0082) and the Health Research Authority (IRAS ID 212761). This study is also registered with the Northern Ireland Hub for Trials Methodology Research SWAT Repository (SWAT 97) (Martin-Kerry et al., 2017).

## Results

A total of 109 participants were randomised, of whom 55 received the ISP and 54 received the MMI resources. Participants were randomised between 25 June 2019 and 17 March 2020.

**Figure 1.**
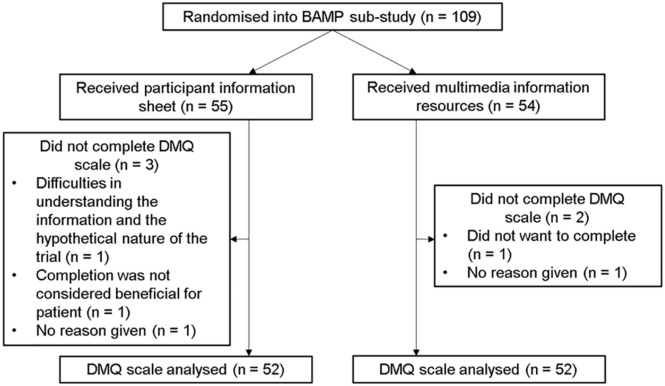
Trial CONSORT diagram.

Five participants (4.6%) did not complete any questions on the DMQ scale and could not be included in analyses (Figure 1). The median (IQR) age for all 109 participants was 13 years (2). The median (IQR) age was 13 years (2) in both the MMI and ISP groups. Overall, 54 (49.5%) of the participants were male. There were 29 boys (52.7%) in the ISP group and 25 boys (46.4%) in the MMI group.

In the ISP group, 47 participants completed the DMQ alone, and three completed it with a parent or carer (two did not give an answer). In the MMI group, 50 participants completed it alone, whereas two completed it with a parent or carer. Summaries of responses to each question in the DMQ scale, broken down by TRECA arm, are given in Table 1. Missing data totals include the five participants who did not complete any of the questionnaire.

**Figure 2.**
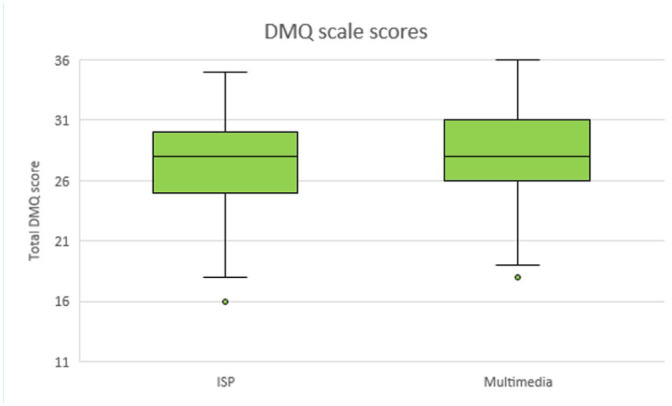
Box plot of total scores on the Decision-Making Questionnaire scale.

Of the 104 participants who completed the DMQ scale, there was only one missing response on items 1–9, and only one response when a participant had circled two adjacent response options. Hence total scores could be calculated for all 104 participants. The overall mean score was 27.5 ±4.3. The mean total scores were higher in the MMI group (28.1 ± 4.2) than in the ISP group (27.0 ± 4.3). The total scores are shown in Figure 2.

The linear regression showed no evidence of a difference between the two groups: AMD 0.99 (95% CI = –0.66 to 2.64; *P* = 0.24). The sensitivity analysis yielded a similar result: AMD 1.06 (95% CI = –0.63 to 2.74; *P* = 0.22).

### Secondary outcomes

#### Individual questions

Comparisons of the item scores from the two trial groups found statistically significant differences on two of the nine items: ‘The information I saw about the BAMP trial was easy to understand’ (Z = −3.03; *P* = 0.003); and ‘I am confident that I have made the right decision about whether or not to take part in the BAMP trial’ (Z = −2.00; *P* = 0.044), both favouring the multimedia information. Among participants who viewed the website, 79.7% rated it as ‘very easy’ or ‘easy’, compared to 54.6% of those allocated to the printed information. ‘Very easy’ ratings were given by 24.1% and 9.1%, respectively. On whether they were confident in their decision-making, 83.3% of those in the website group stated ‘yes, completely’ or ‘yes, mostly’, compared to 72.7% in the printed information group. The differences between the groups on the other seven items were not statistically significant. Results are given in Tables 1 and 2.

**Table 2.** Exploratory analysis of each question in the Decision-Making Questionnaire.

Question	Allocation	N	Median (IQR)	Z-statistic	*P* value
Q1 The information I saw about the BAMP trial was easy to understand.	ISP	52	3 (1)	−3.03	0.002
	MMI	52	3 (0.5)		
Q2 After seeing the information about the BAMP trial I knew what taking part would be like.	ISP	52	3 (1)	−0.13	0.940
	MMI	52	3 (1)		
Q3 The information helped me understand how my treatment or care might change if I took part in the BAMP trial.	ISP	51	3 (0)	−0.52	0.601
	MMI	52	3 (1)		
Q4 The possible benefits of taking part in the BAMP trial were made clear in the information.	ISP	52	3 (1)	−0.53	0.602
	MMI	52	3 (1)		
Q5 The possible disadvantages of taking part in the BAMP trial were made clear in the information.	ISP	52	3 (1.5)	−0.92	0.362
	MMI	52	3 (1)		
Q6 The information about the BAMP trial helped me discuss the trial with the person who asked me to take part (usually a doctor, nurse or researcher).	ISP	52	3 (0)	−0.04	0.981
	MMI	52	3 (2)		
Q7 The information about the BAMP trial helped me discuss taking part with my parent(s) or family.	ISP	52	3 (1)	−0.50	0.647
	MMI	52	3 (0)		
Q8 I am confident that I have made the right decision about whether or not to take part in the BAMP trial.	ISP	52	3 (0.5)	−2.00	0.044
	MMI	52	3 (1)		
Q9 In all, the information about the BAMP trial helped me make my decision about whether or not to take part.	ISP	52	3 (1)	−1.41	0.160
	MMI	52	3 (1)		

BAMP, bone anchored maxillary protraction; ISP, participant information sheet; MMI, multimedia website.

#### Free-text responses

Of the 104 respondents, 63 (60.6%) made at least one positive comment about the information (34 in the MMI group, 29 in the ISP group). The difference between groups was not statistically significant (odds ratio [OR] = 1.50; 95% CI = 0.68–3.30; *P* = 0.32). Three respondents made negative comments about the information (one in the MMI group and two in the ISP group).

In answer to question 10, ‘Was there anything you wanted to know about the BAMP trial but which wasn’t included in the information you saw?’, 15 participants (14.4%) replied ‘yes’ (eight in the MMI group, seven in the ISP group), although 17 participants provided responses (nine MMI, eight ISP) (Table 3; Supplementary data). In the ISP group, six respondents would have liked more information on a variety of aspects, including three who wanted to know more about possible harms of treatment. One would have liked the inclusion of images to aid understanding. In the MMI group, the responses were similarly varied.

To question 11, ‘Can you tell us which aspect(s) about BAMP was explained well in the information you saw?’ 64 participants (61.5%) responded: 34 in the MMI group and 30 in the ISP group. Responses were highly varied. In the ISP group, five participants mentioned the benefits and disadvantages of the surgery, while another three mentioned its potential benefits. Five participants commented on the description of the process; that is, what would happen. One made a very negative comment about the ISP (‘*It is a lot of writing and not attractive to read and it is boring*’). In the MMI group, 12 participants mentioned the description of the process, while four mentioned the advantages and disadvantages. Five participants mentioned the videos as being helpful, and one praised the MMI ‘*interface*’.

To question 12 (‘any other comments?’), there were two responses in the ISP group (both negative: ‘*I think it isn’t very attractive*’; ‘*As a mother I feel this is a lot of information for his age group*’) and there were eight responses in the MMI group, of which six made positive comments (‘*the information was explained clearly. . .*’; ‘*very understandable. . .*’; ‘*helps me understand the BAMP trial*’; ‘*the vodcast / cartoons were useful*’; ‘*easy to navigate and understand*’; ‘*helpful when trying to understand how the trial helps*’), with one negative comment (‘*the main reason of the trial was hard to understand*’) and one question.

## Discussion

The DMQs were completed by more than 95% participants and evaluations of the information were mostly positive in both groups. The mean scale score was higher in the MMI group, but the difference was small and not statistically significant. Participants in the MMI group were also more likely to rate the information as ‘easy to understand’ and more likely to state confidence in their decision-making. In the free-text responses, more positive and fewer negative comments were made about the multimedia than the printed information.

The trial had good methodological qualities: random and concealed allocation, as well as good completion rates for the outcome variables. However, its sample size was small, resulting in high levels of variance. Participants were adolescents in the target age range of the actual BAMP trial, although they were making judgements about a trial that was, for them, hypothetical; it may have been difficult for such young participants to imagine themselves in this situation, which would reduce data validity We could have designed the trial so that participants viewed both formats of information (printed and multimedia), to draw direct comparisons; this may have produced more critical, discerning evaluations. However, participants were viewing the information while awaiting treatment and so had limited time. We did not evaluate information retention, nor did we observe or evaluate participants’ use of the information.

The use of multimedia information for trial recruitment remains in its infancy, and there has been relatively little evaluation of this innovative format of delivery; this is especially the case for trials recruiting child or adolescent participants. For example, a systematic review of 20 trials of multimedia information to inform research consent decisions included 10 in which multimedia resulted in better comprehension of the research than printed information (Palmer et al., 2012). Furthermore, in six trials there was evidence of enhanced information retention from multimedia. Notably none of these trials involved children or adolescents. However, in other primary data studies involving children or adolescents, multimedia was more effective than print in three studies (Carr et al., 2012; O’Lonergan and Foster-Harwood, 2011; Tait et al., 2012b) but no more effective in one (Shani et al., 2003).

Multimedia to inform patients about healthcare interventions has been evaluated in a number of settings, and four systematic reviews report benefits, when compared to printed or spoken information: on patient knowledge, condition self-management, satisfaction with care, as well as some clinical outcomes including pain and anxiety (Ciciriello et al., 2013; Dahodwala et al., 2018; Dekkers et al., 2018; Knox et al., 2019; Tuong et al., 2014). However few of the included primary studies involved child or adolescent patients, and none has involved orthodontics or dentistry. In child or adolescent populations, there has been more evaluation of video animations (which were a component of the TRECA MMI). For example, in children with epilepsy the provision of animated video information had positive impacts on knowledge and medicine adherence, and in studies involving children with respiratory conditions animations had positive impacts on use of medication delivery devices (Fremont et al., 2018; Indradat et al., 2014; Saengow et al., 2018).

The results of this small trial, using a hypothetical scenario, show that multimedia information (such as a website) can provide information to potential trial participants at least as well as printed information, and it may improve ease of understanding, decisional confidence and other subjective evaluations. This is consistent with studies of children’s and adolescents’ use of health technologies, which have emphasised the crucial importance of the included language (Garrido et al., 2019). Participants’ evaluations of the MMI were mostly positive. It was notable that no concerns were expressed about privacy and confidentiality associated with the online information, as these concerns have been prominent in other research (Blower et al., 2020). However, this was a hypothetical study setting for these participants and the MMI did not require users to input personal information.

Multimedia offers a choice of information format to users and potentially allows them to more easily and preferentially access content that is important to their decisions on research participation or healthcare. Enhanced interest and engagement can lead to improved understanding and retention. However, there remains a lack of research with children and adolescents, whose preferences and needs may be different to adults. Furthermore, reporting of research in this area is often less helpful than it might be: what comprises ‘multimedia’ varies greatly among studies (in particular historically) although its description in publication is often brief (Ciciriello et al., 2013; Dahodwala et al., 2018; Dekkers et al., 2018; Knox et al., 2019; Palmer et al., 2012; Tuong et al., 2014).

The multimedia resources in the TRECA study are currently being evaluated in six recruitment SWATs (Martin-Kerry et al., 2017), which will indicate their effects on quality of decision-making and actual trial participation rates. Multimedia offers great promise in the delivery of information in this setting, but careful development, evaluation and reporting are crucial to ensure that resources are suitable and useful.

## Conclusion

Adolescent orthodontic patients found hypothetical trial information conveyed on a website easier to understand and they also had more confidence in their decision-making, compared to those who read printed information. The website information also received more positive and fewer negative evaluations.

## Supplemental Material

sj-doc-1-joo-10.1177_14653125211024250 – Supplemental material for Evaluating the use of multimedia information when recruiting adolescents to orthodontics research: A randomised controlled trial Click here for additional data file. Supplemental material, sj-doc-1-joo-10.1177_14653125211024250 for Evaluating the use of multimedia information when recruiting adolescents to orthodontics research: A randomised controlled trial by Peter Knapp, Nicky Mandall, Wendy Hulse, Jenny Roche, Thirimon Moe-Byrne, Jacqueline Martin-Kerry, Rebecca Sheridan and Steven Higgins in Journal of Orthodontics

sj-docx-1-joo-10.1177_14653125211024250 – Supplemental material for Evaluating the use of multimedia information when recruiting adolescents to orthodontics research: A randomised controlled trial Click here for additional data file. Supplemental material, sj-docx-1-joo-10.1177_14653125211024250 for Evaluating the use of multimedia information when recruiting adolescents to orthodontics research: A randomised controlled trial by Peter Knapp, Nicky Mandall, Wendy Hulse, Jenny Roche, Thirimon Moe-Byrne, Jacqueline Martin-Kerry, Rebecca Sheridan and Steven Higgins in Journal of Orthodontics
