# Supplementary material for: Evaluating the use of multimedia information when recruiting adolescents to orthodontics research: A randomised controlled trial
Source: J Orthod. 2021 Jul 6;48(4):343–51. doi: 10.1177/14653125211024250 (PMC8652365; doi:10.1177/14653125211024250)
Supplement: sj-doc-1-joo-10.1177_14653125211024250 – Supplemental material for Evaluating the use of multimedia information when recruiting adolescents to orthodontics research: A randomised controlled trial [file sj-doc-1-joo-10.1177_14653125211024250.doc]

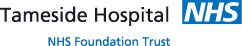


Tameside General Hospital

Ashton-Under-Lyne

OL6 9RW

Telephone 0161 331 6000

**Bone Anchored Maxillary Protraction (BAMP) RCT**

**Patient Information Sheet**

**Version 2: 05/02/2015**

We would like to invite you to take part in a research study. The following information explains all about our research and why it is being done. This is so you understand what we are asking you to do. Please take time to read the information carefully. You can ask us questions or talk to other people about the study if you wish.

Part 1 tells you the purpose of this study and what will happen if you take part. Part 2 gives you more detail about how the study is carried out. Ask us if there is anything that is not clear or if you would like further information. Take time to decide whether or not you wish to take part.

**PART 1**

WHAT IS THE PURPOSE OF THE STUDY?

We are asking people like you to take part because your top jaw and teeth are set back behind your bottom teeth. This is called a reverse bite. As you will know, orthodontic braces alone cannot correct the gap between your top and bottom teeth. Usually, there is no surgery that can be done to your jaw before you are 17 years old. But now there is a new operation that can be offered to the 11- 14 years old age group. The idea of the study is to look at how good the new operation is in correcting a reverse bite.

The way we are looking at the new operation is called a “randomised trial”. Sometimes we don’t know which way of treating patients is best so we are looking at the results of the surgery compared with having no surgery. Each patient is put into a group by chance (randomly). You have an equal chance of being put into group 1 (surgery) or group 2 (no surgery).

WHY HAVE I BEEN INVITED?

You have been asked to take part in the study because you have a reverse bite and you are between 11 and 14 years old. Every patient who has a reverse bite like you is being asked to help. Across the U.K., there are many hospitals taking part and there will be 60 patients in the study.

DO I HAVE TO TAKE PART?

No, you do not have to take part in the study if you do not want to. It is up to you to decide. We will go through this information sheet with you and then leave it with you to read. You will be asked to sign a consent form to show you have agreed to take part. You are free to withdraw at any time, without giving a reason. If you opt out of the study, it will not affect the standard of care you receive.

WHAT WILL HAPPEN TO ME IF I TAKE PART?

If you sign up to take part, you will be randomly put into the “surgery” group or the “no surgery” group

The study information we need can be obtained from the information collected during your routine visits, such as models of your teeth, face and teeth photographs and face X- rays.

If you are in the “surgery” group, we need to glue 4 small metal brackets on your side lower teeth like a tiny fixed brace. This is done by the orthodontist and a small wire is placed each side. This helps protect the lower surgical hooks so you don’t bite on them. Then the surgery hooks are put in your mouth while you are asleep and this takes about 30 minutes. You go home the evening of your operation and will need about 1 week off school. Six weeks after the operation, we ask you to wear elastics in your mouth to move your top jaw and teeth forwards.

If you are in the “no surgery” group you do not have to do anything. We still see you each year to see how your jaw is growing.

All we would like to do for everyone taking part is to take measurements, x-rays of your jaws and models of your teeth so that we can see how they move over the next 3 years.

Additional information over and above routine care:

There are some short questionnaires about how you feel about your face and teeth which take 5 -10 minutes to fill in. We would also ask you to fill in a discomfort score, once a day, for two weeks after your operation if you are in the surgery group. All patients may have two additional jaw X rays over and above the usual face X-rays. We given more detail about this later in this section.

We will also ask you to tell us your opinion of the treatment and result. A check of your hospital records would be made for information such as how long your operation takes.

WHAT WILL HAPPEN IF I DO NOT TAKE PART?

If you do not want to take part in the study, that is fine. We normally see you when you are 16- 17 years old to offer the standard jaw surgery to correct the reverse bite. When you are 16 years old you can decide if you wish to have this jaw surgery or not.

WHAT DO I HAVE TO DO?

All you have to do is sign the consent form to take part in the study. You will then come along for your usual hospital appointments. We will then ask you to complete some questionnaires about how you feel about your teeth and jaws. You do not have to do anything extra except complete the questionnaires. If you are in the “surgery” group we will also ask you to fill in the discomfort scores each day for two weeks after your operation.

WHAT IS THE PROCEDURE THAT IS BEING TESTED?

We are looking at using small metal attachments inside your mouth so we can attach elastics from your top jaw to your bottom jaw. The elastics pull your top jaw and teeth forwards to correct your reverse bite. The metal plates attach behind your upper molar teeth on both sides and also near your bottom front teeth on both sides. A little hook can be seen next to your teeth and you attach the elastics to the hooks at home. The elastics are changed every day. You can still open your mouth, speak and eat with the elastics in position.

WHAT ARE THE SIDE EFFECTS AND POSSIBLE DISADVANTAGES/RISKS OF TAKING PART?

If you are in the “surgical” group there are risks of surgery that we warn everyone about. The risks of the operation will already have been explained to you. If you are in the “no surgery” group there are no risks to you.

As part of this study you may have two X-rays of the jaw over and above the usual face X-rays. One of these is to help plan your operation, if you are having one. The other additional jaw X-ray is taken 1 year after you sign up for the study. X-rays are a type of ionising radiation. You are exposed to natural sources of radiation from the environment all the time. The amount of radiation that patient having braces followed by a jaw operation receives is equivalent to less than a week of this environmental radiation. The extra X –rays, mentioned above, are considered to be a minimal risk by the medical radiation experts.

WHAT ARE THE POSSIBLE BENEFITS OF TAKING PART?

There are no direct benefits to you by taking part in our study apart from the general benefits of the surgical treatment you may have. However, the information we obtain from the study will help improve the future treatment of people with reverse bite. It will help orthodontists and surgeons to decide on the timing of surgery for other teenagers with reverse bite.

WHAT IF THERE IS A PROBLEM?

Any complaint about the way you have been dealt with during the study, or any possible harm you might suffer, will be addressed. The detailed information on this is given in Part 2.

WILL MY TAKING PART IN THIS STUDY BE KEPT CONFIDENTIAL?

Yes. We will follow ethical and legal practice and all information about you will be handled in confidence. The details are included in Part 2.

If the information in Part 1 has interested you and you are considering taking part, please read the additional information in Part 2 before making any decision.

**PART 2**

WHAT IF RELEVANT NEW INFORMATION BECOMES AVAILABLE?

Sometimes new information comes out about the treatment being studied. If this happens, we will tell you and discuss whether you should complete the study. If you decide not to carry on, we will make arrangements for your care to continue. If you continue in the study we may ask you to sign an updated consent form. If the study is stopped for any other reason, we will tell you and arrange your continuing care.

WHAT WILL HAPPEN IF I DON’T WANT TO CARRY ON WITH THE STUDY?

If you opt out of the study, we will still carry on with any routine treatment. We would still need to use information from your records for the study up to that date. We would check with you first and ask you to sign another consent form.

If you are in the “surgery” group and you decide to stop treatment for any reason, we may still ask you to come in for review appointments. We still collect study information e.g. photographs and models of the teeth.

WHAT IF THERE IS A PROBLEM?

If you have a concern about any aspect of this study, you should ask to speak to the researchers, who can try their best to answer your questions. If you remain unhappy and wish to complain formally, you can do this through the NHS complaints procedure. Details can be obtained from your hospital.

In the event that something goes wrong and you are harmed during the research because of someone’s negligence then you may have grounds for legal action compensation against your hospital NHS Trust . You may have to pay your legal costs. The normal National Health Service complaints mechanisms will still be available to you.

WILL MY TAKING PART IN THE STUDY BE KEPT CONFIDENTIAL?

The information that we keep about your teeth and face would be collected on paper and then entered onto a computer with a study reference number. Therefore, your name and address will not be used and no one will know that you are helping us with our study. No one outside the research study will see any of the measurements that we take on your teeth and jaws and no-one outside the research study will be allowed to look at your hospital notes. Your hospital records will not be used anywhere in any publication.

The paper data will be kept in a locked filing cabinet in a locked room and the computer data is kept on a password protected computer in a locked room. The person responsible for data storage will be Dr Nicky Mandall – Tameside Hospital NHS Foundation Trust. The data will be kept securely for 15 years after the study finishes and then disposed of securely. The information will not be used for any future studies other than this one. The only people with access to the data will be the clinical researchers taking part in the study, a research assistant and a statistician from the University of Manchester.

INVOLVEMENT OF YOUR GENERAL DENTAL PRACTITIONER

We would normally write to your Dentist to let them know you are taking part in a study. You will be asked to sign on the consent form to allow us to inform your Dentist. No study information about you will be sent to your Dentist, it is just to let them know you are helping us.

WHAT WILL HAPPEN TO THE RESULTS OF THE RESEARCH STUDY?

At the end of the study all the information is put together and we write a report. This will be sent to an orthodontic or oral surgery journal so that other orthodontists and surgeons can read about what we have found out. At no point will you be identified in any report. A summary of the study results will also be available to patients who have helped us with our research.

WHO IS ORGANISING THE RESEARCH?

The study is being co-ordinated by Dr Nicky Mandall who is a Consultant Orthodontist at Tameside Hospital NHS Foundation Trust in Manchester, U.K. Your hospital Consultants in Orthodontics and Oral surgery are not being paid to carry out this study or to look after patients during the trial.

WHO HAS REVIEWED THE STUDY?

All the research in the NHS is looked at by an independent group of people, called a Research Ethics Committee to protect your safety, rights, wellbeing and dignity. This study has been reviewed and given a favourable ethical opinion by NRES Committee North West – Greater Manchester Central Research Ethics Committee.

CONTACT FOR FURTHER INFORMATION:

If you would like to ask more specifically about this research project you can contact:

Study Co-ordinator: Dr Nicky Mandall Tel: 0161-922-6461.

If you would like general impartial advice about whether to participate you can contact your local hospital Patient and Advice and Liaison Service (PALS)

If you are unhappy about the study contact: The clinical departmental Manager in Oral surgery and Orthodontics: Tel: 0161-922-6383

Thank you very much for taking time to read about the research. If you agree to take part, you will be given a copy of the information sheet and signed consent to keep.
