# Supplementary material for: Evaluating the use of multimedia information when recruiting adolescents to orthodontics research: A randomised controlled trial
Source: J Orthod. 2021 Jul 6;48(4):343–51. doi: 10.1177/14653125211024250 (PMC8652365; doi:10.1177/14653125211024250)
Supplement: sj-docx-1-joo-10.1177_14653125211024250 – Supplemental material for Evaluating the use of multimedia information when recruiting adolescents to orthodontics research: A randomised controlled trial [file sj-docx-1-joo-10.1177_14653125211024250.docx]

Table 3 (Supplementary data): Summary of free text question responses

|  | **ISP arm** | **Multimedia arm** |
| --- | --- | --- |
| **Q 10a) Was there anything you wanted to know about the BAMP trial but which wasn’t included in the information you saw?** | Yes: 8  No: 28  Answer left blank: 16 | Yes: 7  No: 30  Answer left blank: 15 |
| **Q 10b) If yes, please write them here** | 8 participants responded:  - How long it will take to see change/progress with the surgery.  - I would have liked to see pictures of what it would look like afterwards because I couldn't understand it.  - You could expand more on the disadvantages and the risks of the performance.  - The before and after of having this treatment to prove it is all worth it.  - I would have liked to know how high the possibilities of the procedure going wrong would be and if it did go wrong how it could go wrong if they knew.  - Could the metal on your teeth cut or make ulcers on the side of your cheek  - I don't understand what is the BAMP trial for.  - No, all of the information was good and all that I knew. | 9 participants responded:  - How long would the procedure take, what does BAMP stand for.  - It made kids understand it by it being online.  - What happens if you don't take the trial and it's best you did do it?  - I would like to know if the BAMP is studied anywhere else.  - Can the operation ever make anything worse or will it only get better?  - The details of the whole procedure and the long term effects  - I don't understand why some get online questionnaires and others get paper ones.  - How long the operation takes.  - What would happen if you don't get braces? |
| **Q 11) Can you tell us which aspect(s) about the BAMP trial was explained well in the information you saw?** | 30 participants responded.  (22: answer left blank).  - What it was, advantages and disadvantages.  - What it was about.  - That it would be confidential.  - I think all the information in the second and first part is helpful and useful to anyone who would read it.  - About having a reverse bite.  - How confidential it is and why I was specifically chosen to take part.  - Having the surgery or not having the surgery. Group one and group two.  - The aspect of knowing what will happen and the benefits were explained well.  - It talked about what could happened and they made sure that if you were unhappy then you know what to do.  - The advantages and disadvantages.  - If you don't want to take part at 11-14 years old, then when you are 16-17 you get a jaw surgery.  - It explained well about the purpose of the study and the risks of it.  - Everything but the possibility of it going wrong.  - It explains clearly the advantages and disadvantages of both trials.  - I think they explained that your information will not be shared well.  - You explain about the benefits of taking part and what will happen if your in the surgery group.  - All good.  - All of the benefits and disadvantages that you may face.  - Benefits of the trial and why we are taking part in this trial.  - The aspects that were explained well were the advantages and what happens.  - The disadvantages or risks as it is showing what are the risk and the disadvantages. It also shows what to do such as if you don't want to do it and when you do want to do it and it tells you what would happen for both things.  - All of it.  - It is a lot of writing and not attractive to read and it is boring.  - They explained what it was.  - The info that was explained was where they showed us that people have reverse bite.  - What would happen was explained quite clearly and what I had to do.  - The trial explained well about the process and different options. It also explained very well that it is completely optional and you don't have to take part in the trial.  - What happens during the study. How personal data is affected, who I can contact for help.  - The braces part was explained there.  - What do I have to do. | 34 participants responded.  (18: answer left blank).  - That teenagers who have reverse bite can have jaw surgery/operation.  - What is being tested? What will happen to me if I take part?  - The elements which were explained well were what the procedure is, who would take part and what would happen afterwards.  - I think it explained what would happen to me if I took part very well.  - When taking part what will happen and how will it feel like and that if I don't want to take part it's okay.  - It was clear what would happen if I were to take part in the operation. It was also clear that I may leave at any time.  - The videos.  - I thought the information was useful.  - What it is and if I had to take part.  - The disadvantages of taking part in BAMP.  - The benefits and risks.  - The random selection and operation.  - Benefits and risks.  - There was lots of information about the trial and what taking part in the trial would mean.  - What it actually is and what the effects are.  - The advantages and disadvantages videos.  - Risks.  - Questions section was particularly informative. Videos were also very helpful.  - An operation can (illegible) when you are (illegible) to and usually wait (illegible) into help improve your reverse bite.  - Viewed videos make it clear and short text was easy to understand.  - The benefits and disadvantages of taking part were well explained.  - How they help with fixing certain problems with the jaw and what can help your teeth via the trial.  - The actual trial was well explained and I could understand what was happening.  - That you can get chosen if you’re lucky but if you not lucky you might not get it done.  - The benefits and the risks.  - How simple it is to live normal life as you did before.  - What is happening? What is being tested? Do I have to take part?  - The fact that the interface and the contrast helps the user understand more.  - About what the trail actually is and why it's being done.  - What was going to happen to the jaw.  - Why is it happening, what is being tested and why I have been asked to take part.  - You have to normally wait until you are 17 but the trial helps you see if you can have one aged 11-14 instead.  - Why is it happening? Because it tells me what will happen if I wanted to take part. And it would help people as well.  - All of them were explained good and I understood them all well. |
| **Q 12) If you have any other comments about the information you were given about the BAMP trial, please write them here.** | 2 participants responded:  - I think it isn't very attractive.  - As a mother, I feel this is a lot of information for his age group. Thank you. | 8 participants responded:  - The information was explained clearly and it would be able to explain to someone who doesn't know what the BAMP trial what it is about and what happens.  - How long does it take?  - It was very understandable and the information is easy to be viewed.  - The information helps me understand the BAMP trial and what it does to help.  - The vodcast /cartoons were useful.  - Some visuals of before and after BAMP would be informative. The website very easily laid out and easy to navigate and understand.  - The information was helpful when trying to understand how the trial helps.  - The main reason of the trial was hard to understand. I think it should be written boldly on the homepage of the website. |
